# Molecular mapping of quantitative trait loci for resistance to early blight in tomatoes

**DOI:** 10.3389/fpls.2023.1135884

**Published:** 2023-05-30

**Authors:** Tika B. Adhikari, Muhammad Irfan Siddique, Frank J. Louws, Sung-Chur Sim, Dilip R. Panthee

**Affiliations:** ^1^ Department of Entomology and Plant Pathology, North Carolina State University, Raleigh, NC, United States; ^2^ Department of Horticultural Science, North Carolina State University, Mountain Horticultural Crops Research and Extension Center, Mills River, NC, United States; ^3^ Department of Horticultural Science, North Carolina State University, Raleigh, NC, United States; ^4^ Department of Bioresources Engineering, Sejong University, Seoul, Republic of Korea

**Keywords:** early blight, heritability estimates, QTL analysis, tomatoes, *Solanum lycopersicum* (L.)

## Abstract

Early blight (EB), caused by *Alternaria linariae* (Neerg.) (syn. *A. tomatophila*) Simmons, is a disease that affects tomatoes (*Solanum lycopersicum* L.) throughout the world, with tremendous economic implications. The objective of the present study was to map the quantitative trait loci (QTL) associated with EB resistance in tomatoes. The F_2_ and F_2:3_ mapping populations consisting of 174 lines derived from NC 1CELBR (resistant) × Fla. 7775 (susceptible) were evaluated under natural conditions in the field in 2011 and in the greenhouse in 2015 by artificial inoculation. In all, 375 Kompetitive Allele Specific PCR (KASP) assays were used for genotyping parents and the F_2_ population. The broad-sense heritability estimate for phenotypic data was 28.3%, and 25.3% for 2011, and 2015 disease evaluations, respectively. QTL analysis revealed six QTLs associated with EB resistance on chromosomes 2, 8, and 11 (LOD 4.0 to 9.1), explaining phenotypic variation ranging from 3.8 to 21.0%. These results demonstrate that genetic control of EB resistance in NC 1CELBR is polygenic. This study may facilitate further fine mapping of the EB-resistant QTL and marker-assisted selection (MAS) to transfer EB resistance genes into elite tomato varieties, including broadening the genetic diversity of EB resistance in tomatoes.

## Introduction

Early blight (EB), caused by *Alternaria linariae* (Neerg.) (syn. *A. tomatophila*) Simmons, once classified within *A. solani*), is a serious threat to tomato-producing areas across the globe and particularly in the Southeast USA (Nash and Gardner, 1988). EB symptoms are typically characterized by the formation of dark necrotic lesions with concentric rings on the leaves. Consequently, blighted leaves are defoliated, which can reduce fruit quality and yield (Basu, 1974; Jones, 1991; Rotem, 1994). Due to a lack of cultivars with efficacious resistance, tomato growers have relied on other control measures, such as field sanitation, crop rotation, cultural practices, and intensive calendar-based fungicide application programs (Gleason et al., 1995; Keinath et al., 1996; Louws et al., 1996). One of the strategies to manage EB in tomatoes is the frequent application of quinone-oxidizing inhibitors (Q_o_I; strobilurins), such as azoxystrobin and pyraclostrobin (a single site mode of action fungicide), or protectant fungicides, such as mancozeb and chlorothalonil (Ivors et al., 2007). In potato fields, a shift of *A. linariae* isolates toward Q_o_I fungicide resistance has been reported due to the F129L mutation (Pasche et al., 2005; Pasche and Gudmestad, 2008), and resistant strains have been confirmed in NC (Inga Meadow, personal communication). In the past decades, three EB forecast systems have been developed and used to curtail the costs of and to optimize disease management (Madden et al., 1978; Pennypacker et al., 1983; Pitblado, 1992; Gleason et al., 1995; Keinath et al., 1996; Louws et al., 1996; Cowgill et al., 2005). Among the disease forecasting systems, Tomato Disease Forecaster (TOM-CAST) was deemed an effective strategy to determine the proper timing of fungicide sprays (Pitblado, 1992).

While the use of fungicides can manage EB, it is preferred to grow a resistant variety to manage the disease. So far, no single-gene conferring resistance to EB has been identified in the cultivated tomato or its wild relatives (Zhang et al., 2003). Although a great deal of effort has been made toward developing tomato cultivars resistant to EB at North Carolina State University (NCSU), only a few moderately resistant lines and cultivars have been identified (Gardner, 1984; Gardner, 1988; Nash and Gardner, 1988; Adhikari et al., 2017). These tomato lines and cultivars exhibited partial resistance to EB under severe epidemics but were either late maturing or low-yielding (Foolad et al., 2002; Zhang et al., 2003). In many cases, resistance to EB in tomatoes has been reported to be a complex trait and controlled by quantitative and partially dominant genes with epitasis (Gardner, 1988; Nash and Gardner, 1988; Gardner and Shoemaker, 1999; Gardner and Panthee, 2012). To resolve these problems, quantitative trait loci (QTL) mapping can serve as a suitable approach to unraveling the genetic control of complex and polygenic traits in segregating populations and can provide valuable information on phenotypic trait–molecular marker associations (Wurschum, 2012).

In the past, different molecular markers have been used to identify QTL for EB resistance and to develop consensus genetic maps in tomatoes. Among these, restriction fragment length polymorphisms (RFLPs), microsatellites or simple sequence repeats (SSRs), and resistance gene analogs (RGAs) have been widely used to identify specific genomic regions associated with resistance to EB (Foolad et al., 2002; Zhang et al., 2003; Chaerani et al., 2007; Adhikari et al., 2017). The development of single nucleotide polymorphisms (SNP) molecular markers (Jiménez-Gómez and Maloof, 2009), which are the most abundant source of variation in the genome for both intragenic and intergenic regions, represents a valuable tool to identify polymorphisms among closely related lines and to develop highly saturated genetic maps (Sim et al., 2012b).

In this study, the 174 F_2_-derived F_3_ (F_2:3_) population, from a cross between the resistant tomato line NC 1CELBR and the susceptible tomato cultivar Fla. 7775, was phenotyped for EB resistance in the field and under controlled conditions in the greenhouse and genotyped with single nucleotide polymorphism (SNP) molecular markers. QTL analysis was performed to identify the putative genomic regions associated with resistance to EB in the tomato.

## Materials and methods

### Plant materials

Tomato breeding line NC 1CELBR was developed at North Carolina State University (NCSU). It is a large-fruited fresh-market tomato line with determinate growth habits and is resistant to EB. The line was developed by multiple crosses involving wild species *S. habrochaites* and *S. pimpinellifolium* (Gardner and Panthee, 2010). Dr. Jay Scott, University of Florida, kindly provided the seed of the susceptible cultivar Fla. 7775. Despite other similar characteristics, contrasting EB reactions in NC 1CELBR and Fla. 7775 provided ideal materials to develop a population for genetic mapping studies. Crosses were made in the fall of 2009 at the Mountain Horticultural Crops Research and Extension Center (MHCREC), (NCSU), Mills River, North Carolina (NC). The F_2_ seeds were produced in the spring of 2010 by selfing the F_1_. Subsequently, 174 F_2:3_ families were developed and used for phenotypic evaluation in the field and greenhouse, SNP marker analysis, and QTL mapping.

### Phenotyping of the F_2_ population in the field in 2011 in Waynesville, NC

To evaluate plants for resistance to EB in the field, the experiment was conducted in 2011. Seeds were planted in 72 cell flats (56 × 28 cm^2^) in potting mix in the first week of May, and transplants at about six weeks from seed were planted. In the first week of June 2011, greenhouse-grown seedlings of the 174 F_2_ and F_1_ hybrid (NC 10175), susceptible controls (Fletcher, NC123S and NC 30P), resistant controls (NC 2CELBR and Mountain Merit), and resistant and susceptible parents (NC 1CELBR and Fla. 7775) were planted at the Mountain Research Station, Waynesville, NC. Spacing was 45 cm between plant-to-plant and 150 cm between row-to-row. The soil was a clay-loam texture, and the natural daylight photoperiod was about 14/10 hr, with temperatures averaging 25-30°C at their high and 14-16°C at their low. This field site was chosen because *A. linariae* inoculum naturally occurs each year almost three weeks after transplanting. Parents and F_1_ were planted as a control to make sure that the disease developed well in the susceptible parent and that the resistant parent was healthy even under high inoculum pressure. No fungicide application was made to control the EB whereas late blight and Septoria leaf spot-specific fungicides were applied to control those diseases by spraying Presidio every week in combination with others as per the fungicide spray guide in NC (Ivors, 2011). Each plant was assessed for EB symptoms six weeks after planting to the field using a Horsfall and Barratt (1945) rating scale of 1 to 11, where 1 indicates no EB symptoms on the leaf surface, and 11 indicates complete defoliation. Humid and warm conditions favor *A. linariae* development, which was conducive to EB development in 2011.

### Phenotyping of the F_2:3_ population in the greenhouse in 2015 in Mills River, NC

Seeds of the 174 F_2:3_ population and resistant and susceptible parents (NC 1CELBR and Fla. 7775) were surface-sterilized and sown in the greenhouse at Mills River. Seeds were sown in 4P soil mixture (Fafard^®^, Florida, USA) in flatbed metal trays in a standard seeding mix (2:2:1 v/v/v) peat moss: pine bark: vermiculite with macro- and micro-nutrients (Van Wingerden International Inc., Mills River, NC) in March 2015. After ten days, seedlings were transplanted to 24-cell flats (56 cm x 28cm). Three plants per genotype were planted with two replications, and the experiment was conducted in a completely randomized design. Plants in the greenhouse study were fertilized using a 20:20:20 ratio of nitrogen, phosphorus, and potassium, respectively. Standard greenhouse pesticide application was used for possible insect and bacterial disease control. A single-spore isolate of *A. linariae* Sorauer collected from naturally infected tomato plants in Hendersonville, NC was used in this study. The fungus was isolated from infected leaf tissues and grown on potato dextrose agar (PDA, 39 g of Difco PDA, Becton, Dickinson and Company, Sparks, MD) in 10-cm Petri dishes and incubated at 23° C under white fluorescent lamps with a 12-h photoperiod. This isolate collected from the field was confirmed as *A. solani* using microscopic examination and PCR-based assays (Gannibal et al., 2014). After 10-12 days, conidia were harvested by flooding the plates with sterile distilled water. The inoculum concentration was adjusted to 1 × 10^7^ conidia mL^-1^ using a hemocytometer. Before inoculation, a drop (~10 µL) of Tween 20 (Polyoxyethylene-20-sorbitan monolaurate) was added to the inoculum suspension to facilitate uniform spore deposition onto leaves. Nine-week-old plants were artificially inoculated using a hand sprayer (R & D Sprayers Inc., Opelousas, LA, USA). After inoculation, plants were placed in the dark for 24 h and covered entirely with white plastic to create a relative humidity of > 95%. Each inoculated plant was scored for EB symptoms using a Horsfall-Barratt rating scheme (Horsfall and Barratt, 1945) at 14 and 21 days after inoculation, as described above. Average disease scores were used to measure resistance to EB and to identify QTL in the greenhouse trials.

### DNA isolation and SNP genotyping

Genomic DNA of young leaf tissues of each parent and individual plant from F_2_ generation was extracted using the DNeasy Plant Mini Kit (Qiagen Inc., Valencia, CA). A NanoDrop (Model ND-2000, Thermo Scientific Inc., Wilmington, DE) was used to quantify each DNA sample. Approximately, 50 ng/µl of DNA was prepared from each sample for SNP genotyping. We used an optimized subset of 384 SNPs markers that were derived from the 7,725 SNP array developed by the Solanaceae Coordinated Agricultural Project (SolCAP) (Sim et al., 2012a; Sim et al., 2012b). The subset of markers was selected based on polymorphism rates among six fresh market tomato accessions, including Fla.7776, Fla. 8383, NC33EB-1, 091120-7, Fla. 7775, and NC 1CELBR. Also, the genetic position in the genome based on recombination (Sim et al., 2012a) and the physical position was considered important selection criteria to ensure genome coverage. These 384 SNPs were analyzed using the Kompetitive Allele Specific PCR (KASP) genotyping platform (LGC Genomics, Beverly, MA).

### Data analysis

The visual illustration of the correlation matrix and principal component analysis (PCA) was done by using the R language v3.2.3 coupled with the RStudio interface v1.0.143 and R packages (“FactoMineR”, “factoextra”, “ggplot2”, “ggplots”, “corrplot”), respectively (R Core Team, 2018; Amanullah et al., 2022). The summary statistics and normal probability plots were calculated using the UNIVARIATE procedure of SAS. The heritability was estimated for each environment by calculating variance components using the ‘ASYCOV’ function in PROC MIXED in SAS (SAS Institute Inc., 2012).

Broad-sense heritability (*H^2^*) was estimated using the following variance components from the F_2_ population (Nyquist, 1991; Falconer and Mackay, 1996):


$$H^{2}=\frac{VG}{VP}=\frac{VA+VD}{VA+VD+VE}$$

Narrow-sense heritability (*h^2^*) was determined using a regression analysis of offspring on parent approach, using data from the F_2_ and F_3_ generations as has been used by Ohlson and Foolad (2015) and as follows (Nyquist, 1991; Falconer and Mackay, 1996):


$$h^{2}=\frac{VA}{VA+VD+VE}=\frac{Cov(F3xF2)}{\sqrt{\left(VF3xVF2\right)}}$$

Where, *H*= broad-sense heritability, *h^2^* = narrow-sense heritability, VG=genetic variance, VP=phenotypic variance, VA = additive variance, VD= dominance variance, VE=error variance, VF_2_ = Variance at F_2_ generation, VF_3_ = Variance at F_3_ generation, and Cov (F_3_xF_2_) = Covariance of individuals at F_2_ and F_3_ generations.

### Linkage map construction of F_2_ and QTL analysis

Of the 384 SNP markers tested, 375 were polymorphic between the two parental lines, NC 1CELBR and Fla. 7775, that were used for genetic map construction (Meng et al., 2015). The linkage map was constructed using JoinMap 4.0 (van Ooijen, 2006). The grouping mode was set as the autonomous limit of detection, the mapping algorithm was used to perform regression mapping (limit of detection > 2.5, recombination frequency< 0.4, and jump = 5) (Asekova et al., 2021). The Kosambi mapping function was used to convert recombination frequencies into map distance (Kosambi, 1943). Independent limit of detection and maximum likelihood algorithms were used for grouping and ordering of markers, respectively. The ordering of the markers within each chromosome was based on the recombination events between the markers. Linkage groups were compared with published tomato linkage maps.

QTL analysis was conducted using windows QTL Cartographer v 2.5 (Wang et al., 2010) software. The Composite Interval Mapping (CIM) method was used with the default parameters (model 6). A backward regression was used to perform the CIM analysis to enter or remove background markers from the model. The walking speed was set at one cM for the detection of QTL. The additive effect and the proportion of the phenotypic variation (R^2^) for each QTL were also obtained using this software. A 1000 permutation option was chosen to determine the likelihood of an odd (LOD) score threshold to identify the presence of QTL in both environments (Li et al., 2007; Li et al., 2008; Meng et al., 2015). We used 5 cM scanning steps for the detection of QTL. The coefficient of variance (*R^2^*-value), the relative contribution of genetic components, was calculated and described as the proportion of genetic variance explained by the QTL out of the total phenotypic variation. QTLs explaining more than 10% of the phenotypic variance were considered major QTLs, and QTLs found in at least two environments were considered to be consistent.

To designate each QTL, the letter ‘*q*,’ followed by an abbreviation of EB resistance (EBR) was used as ‘*qEBR.’* Additionally, each QTL was classified by the chromosome in which a QTL was detected and then categorized by QTL number. Any QTL within a 5 cM distance on the same chromosome was regarded as a single QTL.

## Results

### Phenotypic data analysis

**Figure 1:**
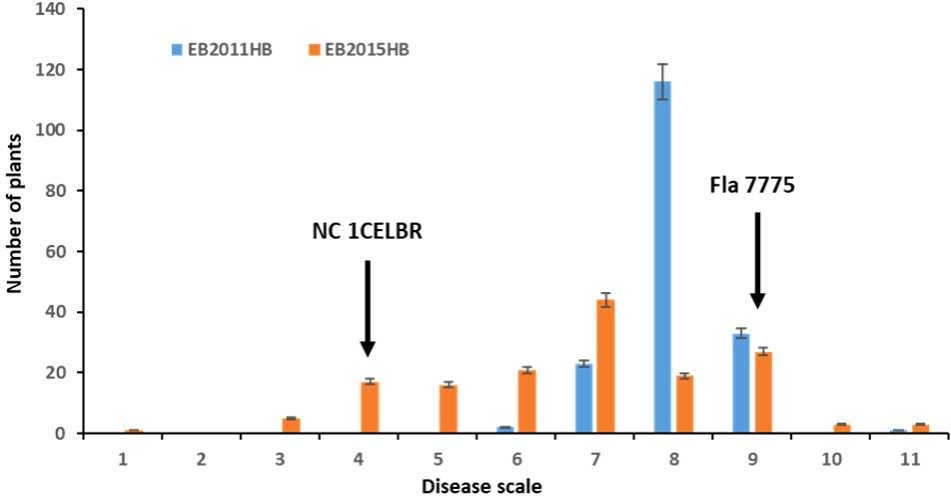
Frequency distribution for disease rating in a population of 174 F_2_ and F_2.3_ progenies. EB2011HB, the F_2_ population was tested in a naturally-infected field at the Mountain Research Station, Waynesville, NC in 2011, and EB2015HB the F_2.3_ progenies were evaluated in an artificial inoculation with a single *A. linariae* isolate in the greenhouse at Mountain Horticultural Research and Extension Center (MHCREC), Mills River, NC in 2015. Each inoculated plant was scored for EB symptoms using a Horsfall-Barratt rating scheme (Horsfall and Barratt, 1945). The values are the means of the parents and progenies, and arrows indicate resistant and susceptible parents. Bars denote the standard deviation.

The disease symptoms of infected tomato plants in the greenhouse experiment varied from chlorotic and necrotic areas of leaves with concentric rings to defoliation and death. The two parental lines exhibited distinguished responses to EB, with NC 1CELBR being consistently resistant (disease score 3.0), and Fla. 7775 being susceptible (disease score 9.0) (**Figure 1**). The inoculated plants were scored for EB symptoms using a Horsfall-Barratt rating scheme (Horsfall and Barratt, 1945) at 14 and 21 days after inoculation. In field experiments, higher disease severities (6 to 11) were observed in 2011 (**Figure 1A**). There was a significant variation among F_2:3_ lines for visual disease rating (**Figure 1** and **Table 1**). Distribution of both field and greenhouse phenotypic data was continuous, indicating quantitative and polygenic control of EB resistance in tomatoes (**Figure 1**).

**Table 1:** Basic statistics of early blight development measured using a Horsfall and Barratt (1945) scale in the tomato population developed from NC 1CELBR × Fla. 7775.

Year	Environment	Sample size	Mean	Standard deviation	Minimum	Maximum	Variance	Heritability (%)
2011	Field (Waynesville)	174	8.1	0.62	6	11	0.36	28.3
2015	Greenhouse (Mills River)	174	6.8	1.7	1	11	9.61	25.3

**Figure 2:**
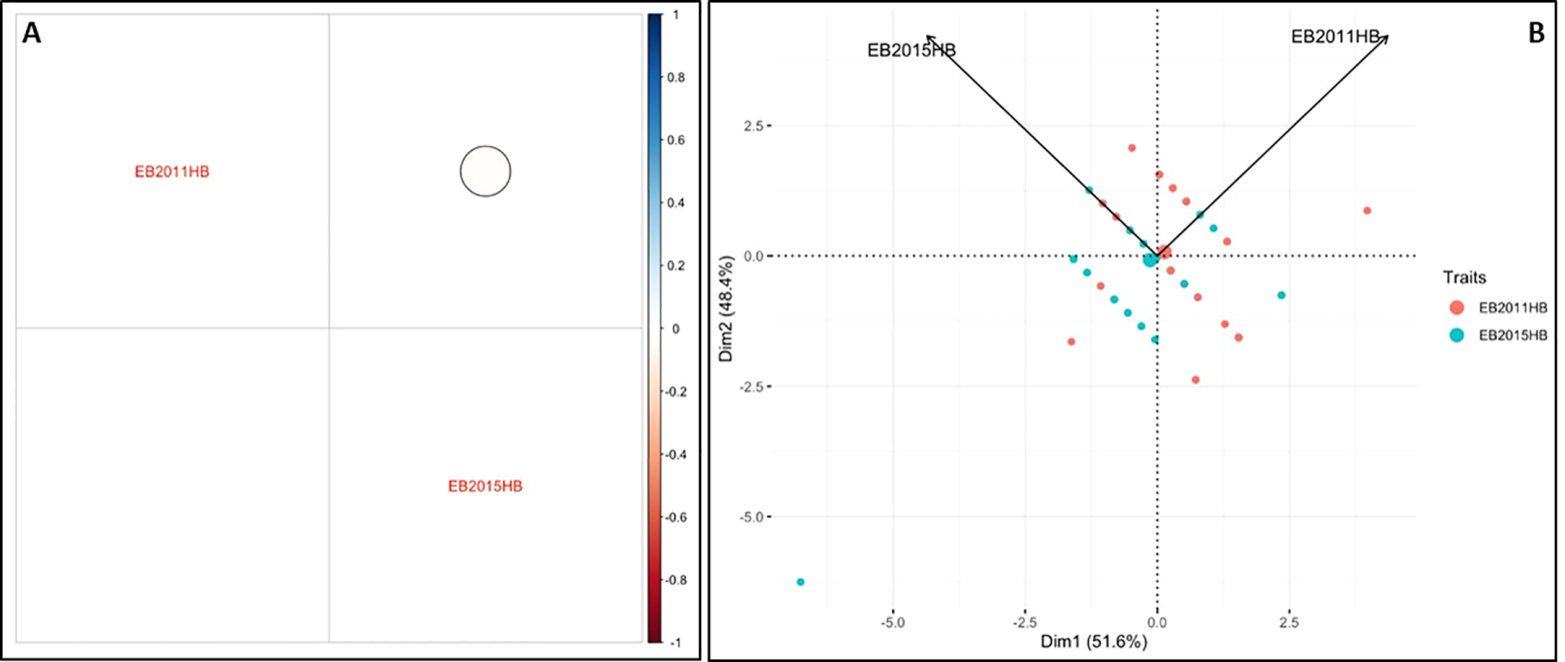
Analysis of phenotypic variability and correlation for early blight resistance in the mapping population. **(A)** Pearson’s correlation between EB2011HB and EB2015HB **(B)** Principal component analysis (PCA) explains the potential phenotypic variability.

The minimum and maximum EB development in 2011 in the population was 6 and 11, respectively, with an average of 8.1. In 2015, the minimum and maximum disease developments in this population were 1 and 11, with an average of 6.8 (**Figure 1** and **Table 1**). These basic statistics over the years indicated that there was a good distribution of EB resistance in this population. The broad-sense heritability estimate for phenotypic data was 28.3%, and 25.3% for 2011, and 2015 disease evaluations, respectively. The disease score values showed a negative correlation between the years 2011 and 2015 (**Figure 2A**). The PCA bi-plot showed the possible association and high percentage of phenotypic variability was observed between the data sets of EB resistance in both environments (**Figure 2B**). The dimension of the first PC (Dim1) broadly outlined and explained 51.8% of the phenotypic variability for EB resistance in 2011 (**Figure 1B**). The dimension of the second PC (Dim2) also distinguished the 48.4% of phenotypic variability for EB resistance in 2015 at opposite angles of the PCA biplot (**Figure 2B**). This data also showed that EB resistance is controlled by multiple genes.

### Linkage map construction of F_2_

**Figure 3:**
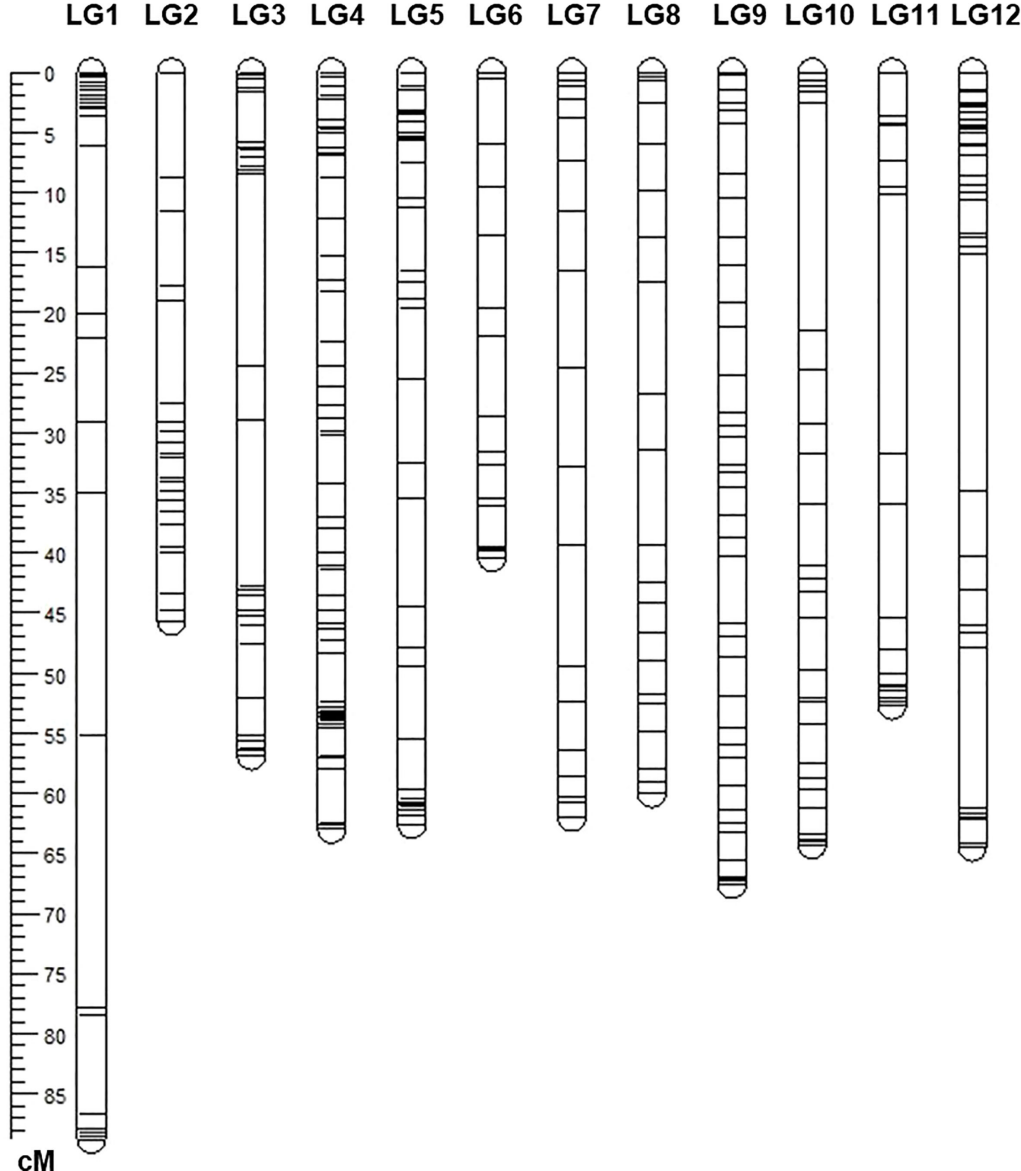
The linkage genetic map of the population of 174 F_2_ progenies. The genetic map was developed from a cross between the resistant tomato line NC 1CELBR and the susceptible tomato cultivar Fla. 7775 using Solanaceae Coordinated Agricultural Project (SolCAP) derived Kompetitive Allele Specific PCR (KASP) markers.

A total of 375 SNP markers were polymorphic between the parents. Those markers were used to genotype the population. A linkage map was constructed with these markers which covered approximately 737.17 cM genetic distance. The map results yielded a total of 12 linkage groups which are comparable with other tomato linkage maps and the number of tomato chromosomes. The Individual chromosomes had 18 to 65 markers with lengths ranging from 42.04 to 88.87 cM (**Figure 3**). Nearly 65 SNP markers were mapped on chromosome 4, followed by 42 SNP markers on chromosome 12 (**Figure 3**).

### QTL analysis

**Figure 4:**
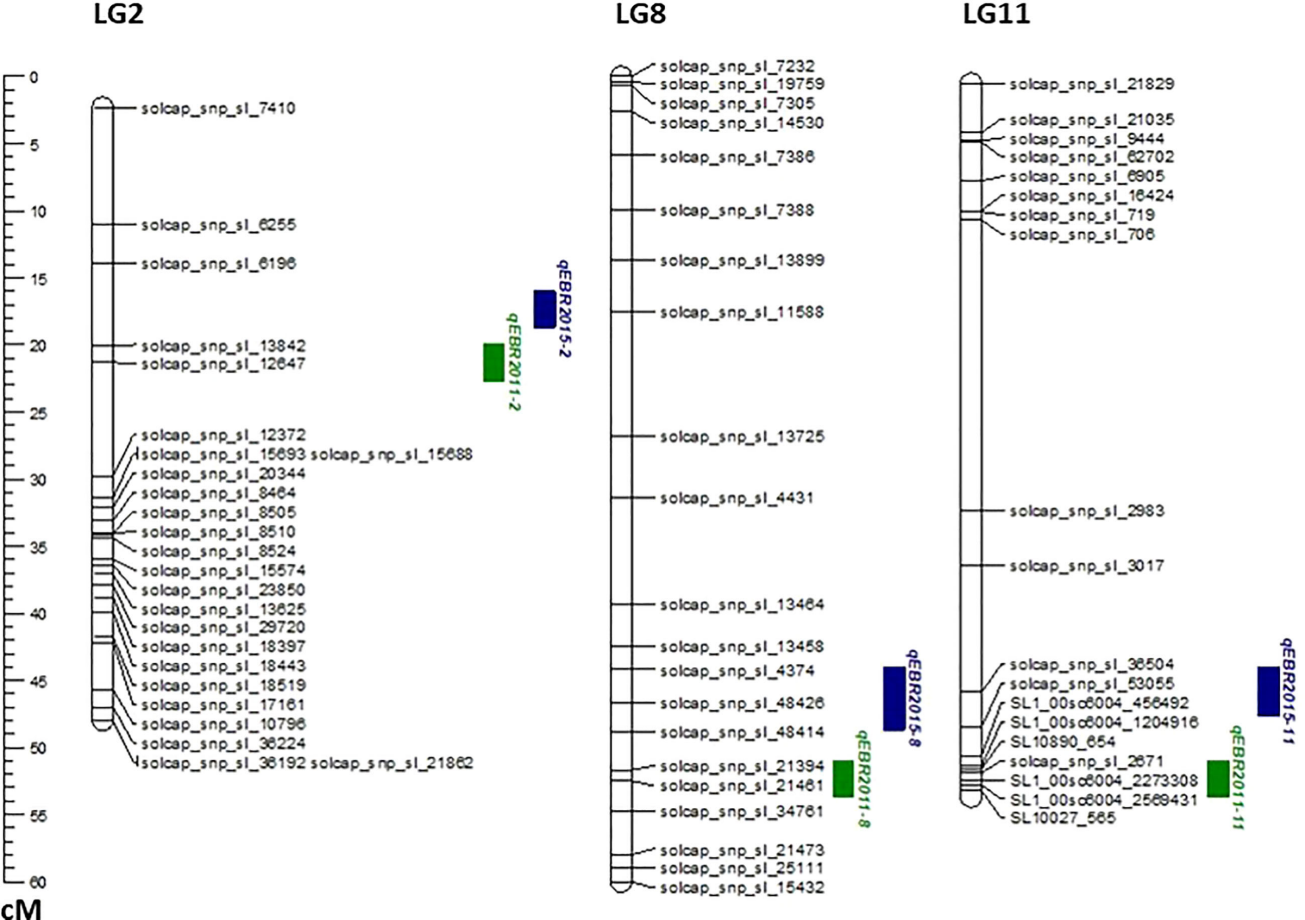
QTL analysis for early blight (EB) resistance in the F_2:3_ mapping populations. Genetic linkage groups showing markers and the locations of EB-resistant QTLs in two different environments with the genetic distance shown in centimorgans (cM) for the mapping population evaluated during 2011and 2015.

**Figure 5:**
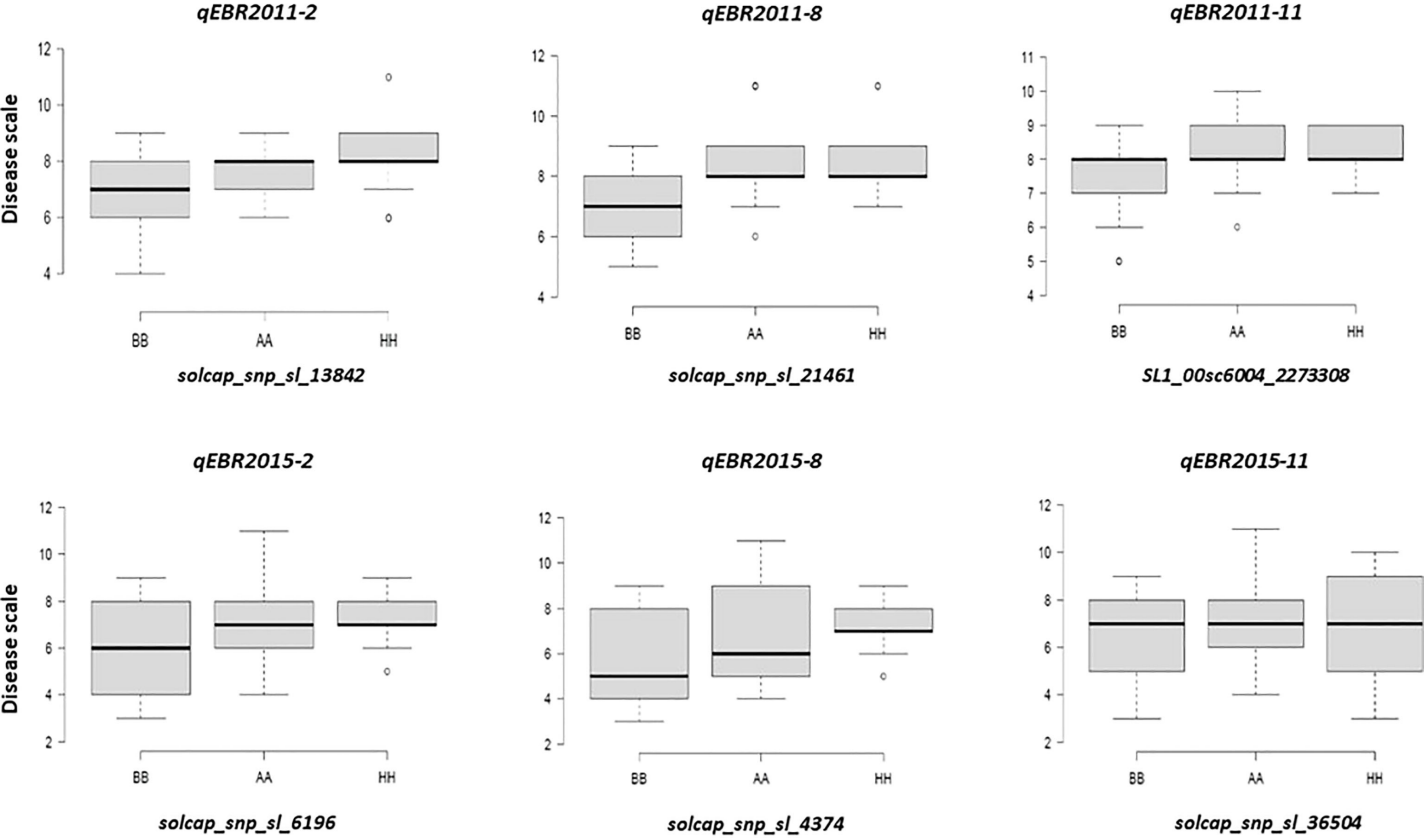
Box plots of resistance level regulated by linked markers to QTLs in F_2_ segregating populations. Genotypes were grouped based on the associated SNP markers. AA: Fla. 7775, BB: NC 1CELBR, HH: Heterozygous.

We identified QTLs for EB resistance using 174 F_2:3_ derived lines and the SNP-based linkage map in two environments (**Figure 4** and **Table 2**). In total, 6 QTLs, including major and minor effects, common for both environments were identified across the genome, explaining phenotypic variation (R^2^) ranging from 3.8 to 21.0% (**Figure 4** and **Table 2**). The QTLs on chromosomes 2, 8, and 11 (*qEBR2011-2*, *qEBR2011-8*, and *qEBR2011-11*) were detected in 2011, respectively. The QTLs *qEBR2011-2* (LOD: 4.2), *qEBR2011-8* (LOD: 4.2), and *qEBR2011-11* (LOD: 4.0) explained 3.8%, 12.1% and 11.7% of total phenotypic variations (**Figure 4** and **Table 2**). The QTLs on same chromosomes were detected in 2015 as well (**Figure 4** and **Table 2**). The QTLs *qEBR2015-2* (LOD: 5.0), *qEBR2015-8* (LOD: 5.2), and *qEBR2015-11* (LOD: 9.1) explained 21%, 11.4% and 19.8% of total phenotypic variations (**Figure 4** and **Table 2**). We used the linked markers of the resistant QTLs to compare the resistance levels and allelic effects in the mapping population (**Figure 5**). As shown in the box plots, the homozygous resistant genotypes BB were associated with enhanced resistance compared to the homozygous susceptible genotype AA for all the QTLs in both environments (**Figure 5**). It also confirmed that all the resistant alleles in mapping population were inherited from NC 1CELBR. These results indicated that multiple genes/QTLs are contributing to EB resistance.

**Table 2:** Quantitative trait loci (QTL) for early blight (EB) resistance in tomato detected by composite interval mapping (CIM) in a population of 174 F_2.3_ progenies.

Trait	QTLs	Linkage group	Position (cM)	LOD	R^2^ (%)	Additive	Dominant
EB2011HB	*qEBR2011-2*	2	20.01	4.17	3.8	-1.42	-2.53
EB2011HB	*qEBR2011-8*	8	51.31	4.18	12.1	-1.44	-2.64
EB2011HB	*qEBR2011-11*	11	50.91	4.03	11.7	-1.44	-2.65
EB2015HB	*qEBR2015-2*	2	16.61	5.02	21.0	0.71	-5.91
EB2015HB	*qEBR2015-8*	8	32.41	5.24	11.4	2.81	3.91
EB2015HB	*qEBR2015-11*	11	44.12	9.11	19.8	-2.19	-5.81

## Discussion

We developed F_2_ and F_2_-derived mapping populations from a cross between the tomato breeding line NC 1CELBR (EB-resistant) and the susceptible tomato cultivar Fla. 7775 (EB-susceptible). The population was assessed for resistance to EB in the field trial and replicated greenhouse trials and genotyped with SNP molecular markers. Both field and greenhouse phenotypic data exhibited continuous distributions. The CIM analysis revealed 6 QTL conferring resistance to *A. linariae*. These QTLs explained up to 21% of the phenotypic variation confirming that genetic control for resistance to EB in NC 1CELBR is polygenic. The discovery of multiple QTL suggested that EB resistance in NC 1CELBR contributed different degrees of resistance to EB and behaved as a quantitatively inherited trait.

The estimate of broad-sense heritability (*H^2^*) was 28.3% in the field test; whereas, in the greenhouse experiments it was 25.3%, suggesting a significant environmental effect on EB development in this mapping population. It is not surprising to have low narrow-sense heritability in this population since the heritability was determined from early (F_2_ and F_3_) generations. If the disease were evaluated at later generations, the level of homozygosity would go up, heterozygosity would go down, and resistance loci would have been fixed. The environmental effect could be minimized, and the genetic effect could be maximized, which is ultimately heritability. Disease severity was high in the 2011 field test, and presumably, this could be due to the dispersal of inoculum in the field, and within the plant canopy and variations in micro-climatic conditions, particularly dew and rain events, that would influence disease development during the tomato growing period (Rotem and Reichert, 1964). To avoid such confounding effects, phenotypic data are likely more reliable when large population sizes or even advanced populations such as recombinant-inbred lines (RILs) are evaluated in different environments with multiple replicates (Gardner, 1990). Nonetheless, we found the F_2_ population had considerable resistance to EB and can be used to advance our effort to develop EB-resistant tomatoes and to combine multiple disease resistance with good fruit quality, which was started by releasing improved breeding lines and hybrids from our program before (Gardner and Panthee, 2010; Panthee and Gardner, 2010). Furthermore, NC 1 CELBR is the first identified tomato line that combines early blight resistance with the *Ph-2* and *Ph-3* genes for late blight resistance. The line was developed by performing crosses comprising wild species *S. habrochaites* and *S. pimpinellifolium* (Gardner and Panthee, 2010; Panthee and Gardner, 2010). It is worthwhile as parents in developing multiple disease resistant F_1_ hybrids as well as parental lines for future tomato breeding programs with joint resistance to late blight and early blight without a linkage drag.

The results suggested that a functionally related QTLs conferring resistance to EB in the field and greenhouse had identical genetic regions. Although the QTLs were identified in the same genetic region, phenotypic variations in disease reaction between the field and greenhouse tests differed. In general, phenotypic variations in the 2011 field trial were lower compared to 2015 greenhouse trial. These results further emphasize that multiple replicated trials are necessary to conduct field EB evaluation and QTL identification. Furthermore, QTL detection is dependent on the level of precise phenotyping. We used foliar disease rating in the present study. Stem lesion was found to correlate better with the level of disease resistance, mainly when experiments are conducted in the greenhouse (Gardner, 1990). Anderson et al. (2021) have reported three QTLs from chromosomes 1, 5 and 9 based on foliar and stem lesions scoring. Therefore, it may be worth using stem lesions as well as foliar symptoms for EB QTL analysis in future studies.

Molecular markers and genetic maps are powerful tools to dissect complex traits and develop marker-assisted breeding strategies in tomatoes (Panthee and Chen, 2010; Foolad and Panthee, 2012). Foolad et al. (2002) developed BC_1,_ and BC_1_S_1_ populations of the *Solanum lycopersicum* x *S. habrachaites* cross and tested these in fields from 1998 - 2000. They identified ten major QTLs for resistance to EB using interval mapping. In another study, Zhang et al. (2003) identified six QTLs, four as major QTLs on chromosomes 5, 8, 10, and 11, and two as minor QTLs on chromosomes 3 and 8. Both previous studies identified QTLs for resistance to EB using RFLP, SSR, and RGA markers (Foolad et al., 2002; Zhang et al., 2003), and they concluded that a high level of similarity between the two field studies was indicative of the stability of QTLs across populations and environments. In the present study, the reported QTLs were found in at least two experiments that were regarded as consistent QTLs as defined above. Although a different mapping population and markers were used, the QTLs detected on chromosomes 8 and 11 in this study agreed with the results of the previous studies (Foolad et al., 2002; Zhang et al., 2003). Ashrafi and Foolad (2015) identified four QTLs that are associated with EB from chromosomes 2, 5, 6, and 9. The positions of the QTLs found in the present study could not be compared because of the different marker types and genetic distance on the map. Furthermore, in the present study, even QTLs were detected at similar locations but the explained phenotypic variations were differ in different environments attributing to the environmental effects. The present study utilized SNP markers to identify QTLs resistance to EB and appeared to be useful for mapping and marker-assisted selection. Although we identified several SNP markers associated with QTLs for resistance to EB, these QTLs are likely to play distinct roles in plant defenses and plant innate immunity. The biological functions of these QTLs or genes in this pathosystem remain a critical unanswered question. Cloning, molecular characterization, and functional analysis of these QTLs in the tomato A*. linariae* interactions deserve further study.

## Conclusion

The NC 1CELBR × Fla. 7775 derived mapping population was used to construct a genetic linkage map and QTL analysis for EB resistance. We detected a total of 6 QTLs, among them all QTLs conferring resistance to EB were inherited from NC 1CELBR. The SNP markers identified in this study are closely associated with putative EB- resistant QTLs and may be involved in host defense responses. To validate these results, additional mapping population development and fine mapping are necessary to determine their resistance spectrum to multiple isolates of *A. linariae*. Developing multiple advanced crosses and pyramiding resistance genes with superior quality is necessary to achieve enhanced resistance to early blight in tomatoes through MAS.

## Data availability statement

The original contributions presented in the study are publicly available. This data can be found here: https://zenodo.org/record/7677766#.Y_qoAXbMJRY.

## Author contributions

TBA implemented the experiment and drafted the manuscript. MIS revised the manuscript, and analyzed the data. FJL revised the manuscript. SCS analyzed the data. DRP conceived the idea, designed the experiment, analyzed the data, and provided resources. All authors contributed to the article and approved the submitted version.
